# Liposomal Formulations in Clinical Use: An Updated Review

**DOI:** 10.3390/pharmaceutics9020012

**Published:** 2017-03-27

**Authors:** Upendra Bulbake, Sindhu Doppalapudi, Nagavendra Kommineni, Wahid Khan

**Affiliations:** Department of Pharmaceutics, National Institute of Pharmaceutical Education and Research, Hyderabad 500037, India; upendra.bulbake@gmail.com (U.B.); dsdoppalapudisindhu@gmail.com (S.D.); nagavendra.kommineni@gmail.com (N.K.)

**Keywords:** liposomes, therapeutics, drug delivery, liposome technology, nanotechnology, clinical trials, marketed products

## Abstract

Liposomes are the first nano drug delivery systems that have been successfully translated into real-time clinical applications. These closed bilayer phospholipid vesicles have witnessed many technical advances in recent years since their first development in 1965. Delivery of therapeutics by liposomes alters their biodistribution profile, which further enhances the therapeutic index of various drugs. Extensive research is being carried out using these nano drug delivery systems in diverse areas including the delivery of anti-cancer, anti-fungal, anti-inflammatory drugs and therapeutic genes. The significant contribution of liposomes as drug delivery systems in the healthcare sector is known by many clinical products, e.g., Doxil^®^, Ambisome^®^, DepoDur™, etc. This review provides a detailed update on liposomal technologies e.g., DepoFoam™ Technology, Stealth technology, etc., the formulation aspects of clinically used products and ongoing clinical trials on liposomes.

## 1. Introduction

The concept of liposomal drug delivery system has revolutionised the pharmaceutical field. Alec Bangham, in 1961 [1], first described liposomes. Since then active research in the field of liposomes have been carried out and their applications are now well established in various areas, such as drug, biomolecules and gene delivery [2]. Liposomes are spherical vesicles characterised by a bilayer of lipids with an internal aqueous cavity. Liposome structural components are phospholipids or synthetic amphiphiles incorporated with sterols, such as cholesterol, to influence membrane permeability. Thin-film hydration is the most widely used preparation method for liposomes, in which lipid components with or without a drug are dissolved in an organic solvent. The solvent will be evaporated by rotary evaporation followed by rehydration of the film in an aqueous solvent. The other methods include reverse-phase evaporation, freeze-drying and ethanol injection [2]. Techniques like membrane extrusion, sonication, homogenization and/or freeze-thawing are being employed to control the size and size distribution. Liposomes can be formulated and processed to differ in size, composition, charge and lamellarity.

**Figure 1:**
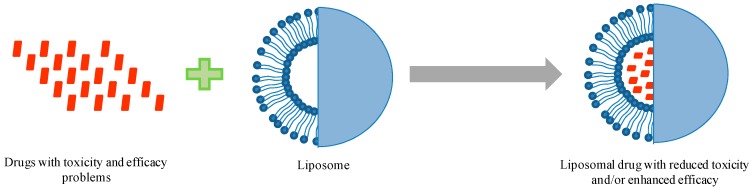
Schematic representation showing the advantages of formulating drugs in liposomes.

**Figure 2:**
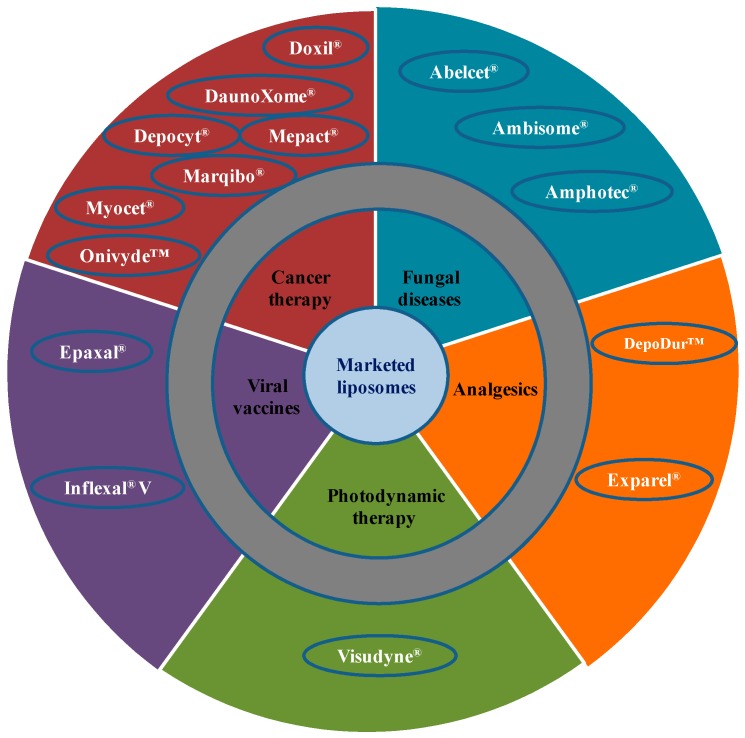
Therapeutic areas covered by liposome-based products.

Due to extensive developments in liposome technology, a number of liposome-based drug formulations are available for human use and many products are under different clinical trials. Encapsulation of drugs in liposomes (as shown in Figure 1) enhanced the therapeutic indices of various agents, mainly through alterations in their pharmacokinetics and pharmacodynamics. Drugs with different solubility can be encapsulated in liposomes, hydrophobic drugs have affinity to the phospholipid bilayer and hydrophilic drugs are entrapped in the aqueous cavity. The first successful milestone in liposome-based products was the introduction of Doxil^®^ to the U.S. market in 1995 for the treatment of patients with ovarian cancer and AIDS-related Kaposi’s sarcoma after the failure of prior systemic chemotherapy or intolerance to such therapy. Gabizon and Barenholz commenced the development of Doxil^®^ in Israel and the USA [3]. It was the first nano-sized liposomal product to obtain regulatory approval. Later, NeXstar Pharmaceuticals USA also developed a liposomal product, DaunoXome^®^, for the delivery of daunorubicin (DNR), which was approved by the U.S. FDA in 1996 for the management of advanced HIV-associated Kaposi’s sarcoma. Subsequently, a few more products have become available for the management of various cancers. These products includes Depocyt^®^ by SkyPharma Inc., Myocet^®^ by Elan Pharmaceuticals, Mepact^®^ by Takeda Pharmaceutical and Marqibo^®^ by Talon Therapeutics. Recently, a fluorouracil and leucovorin combination therapy-based product was approved for metastatic adenocarcinoma of the pancreas. This product is marketed as Onivyde™ by Merrimack Pharmaceuticals, Inc. Although cancer was the most widely explored area in terms of clinically approved products of liposomes, liposomal products were also developed for other diseases (Figure 2). For fungal infections, the U.S. FDA approved Amphotec^®^ and Ambisome^®^ in 1996 and 1997, respectively. Treatment of fungal infections has benefited since the development of these liposomal formulations of Amphotericin B (AmB). Also, liposomes have become important carrier systems in vaccine development and interest in liposomal vaccines have markedly increased because of the development of products Epaxal^®^ and Inflexal^®^ V. Both products are developed by Crucell, Berna Biotech for vaccination against hepatitis and influenza, respectively.

This review gives a concise picture of various liposome-based products available in the market and liposome technologies (Stealth liposome technology, DepoFoam™ technology, Thermosensitive liposomes and Non-PEGylated liposomes) involved in the development of these products. Liposome-based products under ongoing clinical trials as monotherapy or in combination with other agents/therapies are also covered.

## 2. Liposome Technologies for Delivery of Therapeutics

This section emphasises the liposome technologies specifically developed for the preparation of clinically used liposome-based products. Each technology offers unique characteristics in an attempt to optimise drug delivery by protecting the unique properties of the therapeutic agent while minimizing its drawbacks.

### 2.1. Stealth Liposome Technology

**Figure 3:**
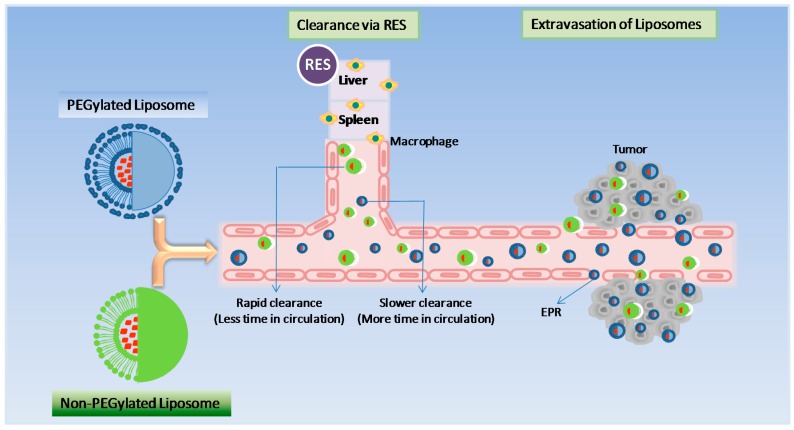
Pharmacokinetics of PEGylated liposomes and Non-PEGylated liposomes.

Stealth technology has been explored in developing a drug delivery system that makes their detection by the mononuclear phagocyte system difficult. In this technology, strands of the polymer(s) are attached to drug molecules or a system that can improve the safety and efficacy of the therapeutic agents. Generally, polyethylene glycol (PEG) is used as a polymer and the process is called PEGylation. In general, PEGylation is attained by the incubation of a reactive derivative of PEG with the target moiety. Covalent linkage of liposome to a PEG protects the active moiety from the recipient’s immune system, which results in reduced immunogenicity and antigenicity. It also produces alterations in the physiochemical properties of the active moiety, including changes in the hydrodynamic size, which further reduce its renal clearance and thereby prolongs its circulatory time (Figure 3). Also, it provides hydrophilicity to hydrophobic drugs and reduces dosage frequency. These changes do not diminish efficacy and show reduced toxicity [4]. Furthermore, due to the leaky nature of the tumour vasculature, nano-sized formulations with prolonged circulatory time show enhanced permeation and retention (EPR) and slowly accumulate in the tumour bed. This technology provided a very successful liposome-based product namely Doxil^®^ as an intravenous injection for the management of advanced ovarian cancer, multiple myeloma and HIV-associated Kaposi’s sarcoma. This technology helps to achieve a customised dosage profile.

### 2.2. Non-PEGylated Liposome Technology

Non-PEGylated liposome (NPL) is a unique drug-delivery system that came as a breakthrough in cancer therapy by offering the benefits of PEGylated-liposome while eliminating the side effects associated with PEG such as hand-foot syndrome (HFS). NPL Doxorubicin (NPLD) injection provides a better safety profile over conventional DOX and Doxil^®^. NPLD not only reduces the cardiac toxicity associated with DOX, but also the dose-limiting toxicity linked with the use of Doxil^®^, such as HFS. This is achieved by a combination of specific composition and a unique manufacturing process of the NPLD liposome, which gives it the desired physicochemical properties. The NPLDs have an increased circulation time and less cardiotoxicity as compared with conventional DOX. Since NPLD do not have a PEG coating, they are not associated with the painful HFS, which is a dose-limiting adverse event with PEG-DOX [5]. Myocet^®^ is a NPLD manufactured by Elan Pharmaceuticals, Princeton, NJ, approved in Europe and Canada for the management of metastatic breast cancer in combination with cyclophosphamide.

### 2.3. DepoFoam™ Liposome Technology

**Figure 4:**
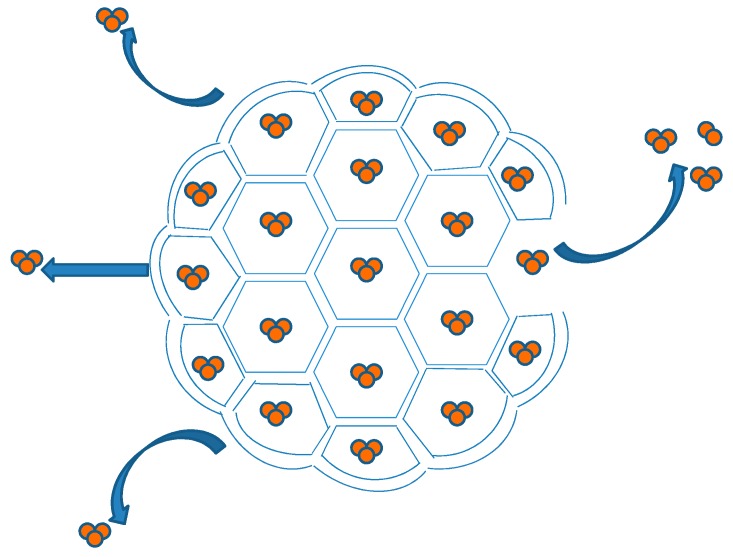
Spheroid and granular structure of a DepoFoam™ particle.

DepoFoam™ is a proprietary, extended-release drug delivery technology introduced by Pacira Pharmaceuticals, Inc., Parsippany, NJ, USA. DepoFoam™ is the core technology behind several marketed products such as Depocyt^®^, DepoDur™ and Exparel^®^. DepoFoam™ technology encapsulates drugs in its multivesicular liposomal platform without modification of their molecular structure. The multivesicular liposomes releases drug(s) over a required period of time ranging from 1 to 30 days. DepoFoam™ consists of microscopic spheroids (3–30 µm) with granular structure and single-layered lipid particles composed of a honeycomb of numerous nonconcentric internal aqueous chambers containing the bounded drug (Figure 4). Each particle contains numerous non-concentric aqueous chambers bounded by a single bilayer lipid membrane. Each chamber is partitioned from the adjacent chambers by bilayer lipid membranes composed of synthetic analogs of naturally existing lipids (DOPC, DPPG, cholesterol, triolein etc) [6]. Upon administration, DepoFoam™ particles release the drug over a period of hours to weeks following erosion and/or reorganization of the lipid membranes. DepoFoam™ technology improved the properties of both small and large molecules. This technology considerably improved patient care by providing a remarkable solution for medications that require frequent multiple injections and have a short period of action or side effects.

### 2.4. Lysolipid Thermally Sensitive Liposome (LTSL) Technology

Thermosensitive liposomes have been studied for drug release at sites of elevated temperature. Generally, lipids, e.g., DPPC, MSPC, with a transition temperature between 40 and 45 °C have been used in the preparation of these liposomes. These novel liposomes are being developed to exhibit temperature-dependent release of encapsulated drug(s). Local tissue temperature is generally elevated to 42 °C by radiofrequency ablation, a technique based on the application of radiofrequency. Lipid components present in the liposome undergo a gel to liquid transition at elevated temperatures, making it more permeable, and thus releasing the drug. Moreover, application of local hyperthermia causes leakage of blood vessels within tumours, thereby increasing accumulation of liposomes in the tumour. ThermoDox^®^ of Celsion Corporation is being tested in phase III clinical trial uses LTSL (lysolipid thermally sensitive liposome) technology to encapsulate DOX for the treatment of various solid tumours. For ThermoDox^®^, this technology allows a 25 times greater concentration of the drug in the treatment area than intravenous (i.v.) DOX. Also, DOX concentration increases significantly in the circulation when compared to other liposomally encapsulated DOX [7].

## 3. Clinically Available Liposome-Based Products

Comprehensive information related to clinically available liposome-based products is provided in this section. Currently, there are good numbers of liposome-based drugs available for human use. Most of the liposomal drug formulations are available for intravenous and intramuscular (i.m.) applications. A brief summary of these products is given in Table 1.

### 3.1. Liposomes for Cancer Therapy

#### 3.1.1. Doxil^®^

Doxil^®^, a formulation containing DOX hydrochloride, is the first FDA-approved nano drug delivery system based on PEGylated liposome technology. Sequus Pharmaceuticals, USA originally developed Doxil^®^ in 1995 as an i.v. injection for the management of advanced ovarian cancer, multiple myeloma and HIV-associated Kaposi’s sarcoma. Single-dose vials of 20 mg/10 mL and 50 mg/25 mL are available and administered at a starting rate of 1 mg/min through i.v. infusion to minimise the risk of infusion reactions.

**Figure 5:**
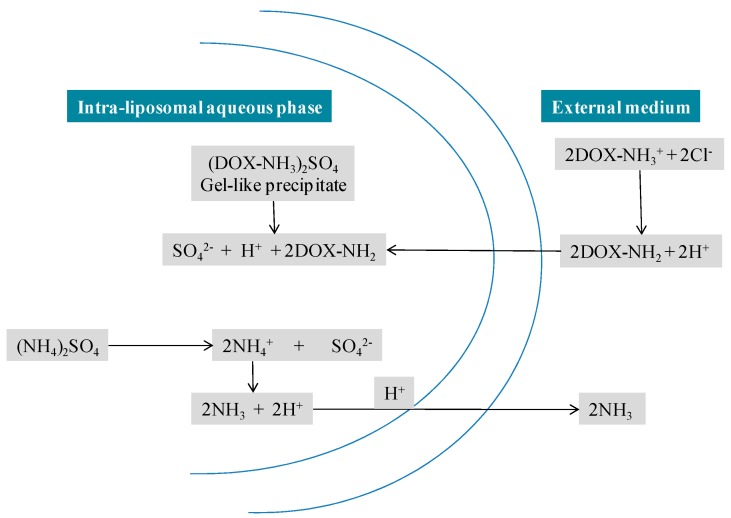
Remote loading approach of DOX into the intraliposomal aqueous phase. Liposomes are prepared at the desired concentration of ammonium sulphate. The gradient was formed by removing the ammonium sulphate from the external liposome medium. Intraliposomal NH_4_^+^ dissociates into NH_3_ and H^+^, NH_3_ escape from the liposome and H^+^ is retained in the liposome water phase. DOX HCl is added to the liposome dispersion at a temperature above the phase transition of the liposomal lipids. DOX, a cationic amphiphile, is present in equilibrium between an ionised and a non-ionised form. The latter form commutes across the liposome bilayer and becomes ionised once exposed to the internal H^+^ environment, and forms a salt with the SO_4_^2−^ anions.

Doxil^®^ liposomes are composed of high phase-transition-temperature (*T*_m_) phospholipid hydrogenated soy phosphatidylcholine (HSPC), cholesterol and *N*-(carbonyl-methoxypolyethylene glycol 2000)-1,2-distearoyl-sn-glycero-3-phosphoethanolamine sodium salt (MPEG-DSPE) in a molar ratio of 56:38:5 [8]. More drug retention was obtained because of the optimum proportion of cholesterol and HSPC, which forms a non-flexible bilayer at 37 °C and below 37 °C. DSPE is incorporated into the bilayer of the liposomes to supply a reactive functional group for the hydrophilic PEG chains (molecular weight 2000) to covalently bind to the DSPE head that elongates into water phases both inner and outer. The overall lipid content of Doxil^®^ is nearly 16 mg/mL and 2 mg/mL is the DOX concentration [9]. DOX is located within the hydrophilic core of the liposomes in the form of DOX–sulphate complex [3]. More than 90% of DOX is encapsulated in the minuscule, unilamellar, vesicular stealth liposomes of 80 to 100 nm size [3]. A high and stable drug/lipid ratio was obtained through the remote loading approach (Figure 5) (first developed by Barenholz [3]) by a transmembrane gradient of ammonium sulphate: [(NH_4_)_2_SO_4_] liposome >> [(NH_4_)_2_SO_4_] medium that serves as a driving force for the efficient and stable loading of amphipathic weak bases into preformed liposomes [10,11]. This remote drug loading approach allows for systematic accumulation of DOX inside the liposome hydrophilic core (about 15,000 DOX molecules/vesicle), with most of the drug (>90%) present as a crystalline-like precipitate that is free from osmotic effects and thus contributes to the stability of the entrapment [12,13]. This approach is based on formulating liposomes that show a transmembrane gradient, which serves as a driving force for the remote loading of amphipathic weak base drugs. This loading technology allows higher retention with less drug efflux in circulation, while providing acceptable rates of drug distribution in tissues [14].

To examine the pharmacokinetics of DOX, Gabizon et al. reported a pilot clinical trial study on 15 cancer patients. In this study Doxil^®^ was compared with the free DOX, at doses of 25 mg/m^2^ and 50 mg/m^2^. This study showed that Doxil^®^ showed a much lower volume of distribution (4 L) compared to the free drug (254 L). Moreover, the clearance of Doxil^®^ (0.1 L/h) was much lower compared to the free drug (45 L/h). Doxil^®^ showed biphasic clearance phase with half-lives of 2 h and 45 h, which shows association of almost all the circulating DOX with the liposome. Doxil^®^ showed 4–16 times higher concentration of DOX in the tumour of patients using the product [15]. Doxil^®^ has shown the ability to clinically reduce cardiotoxicity, a side effect of free DOX treatment, because encapsulated DOX is not bioavailable at cardiac muscle cells and the myocardium [16].

#### 3.1.2. DaunoXome^®^

DaunoXome^®^, a DNR citrate liposomal formulation, is a germ-, pyrogen- and preservative-free product in a single-dose vial for i.v. infusion. NeXstar Pharmaceuticals, USA, developed DaunoXome^®^ in 1996 for the management of HIV-associated Kaposi’s sarcoma [17]. Each single-dose vial contains approximately 50 mg of DNR base enclosed in liposomes composed of 168 mg cholesterol and 704 mg distearoylphosphatidylcholine (DSPC). The aqueous dispersion of these liposomes (25 mL/vial) contains 2125 mg sucrose, 94 mg glycine and 7 mg calcium chloride dihydrate. Liposomes are neutrally charged with a bilayer composed of DSPC and cholesterol at 2:1 molar ratio and a mean particle size of approximately 45 nm. The lipid:drug weight ratio is 18.7:1 (total lipid:DNR base), which is equivalent to 10:5:1 molar ratio of DSPC:Cholesterol:DNR [18]. The specific lipidic formulation of DNR has been shown to form liposomes with excellent physical strength, improving the stability of entrapped DNR from fast metabolisation and also minimising its protein binding [19]. Because of the small size and comparative neutrality of DaunoXome^®^ particles, reticulo-endothelial system (RES) uptake is diminished, leading to extended drug circulation [20].

Liposomal DNR with increasing dosage for safety, pharmacokinetics and potential efficacy in patients with HIV-associated Kaposi’s sarcoma was evaluated by Gill et al. [21]. They found that DaunoXome^®^ at a dose range between 40 and 60 mg/m^2^ was most efficacious. The mean plasma area under the curve (AUC) obtained from these results ranges from 114.91 to 120.1 μg h^−1^ mL^−1^ [21,22]. These results represent an 11–12-fold increase over conventional DNR and reflect the steady nature of the liposomal carrier, as reported in murine model systems [20,21]. Similarly, elimination is significantly low in DaunoXome^®^-treated patients as compared to conventional DNR treated patients (10.5 vs. 233 mL/min, respectively) [22,23]. These two properties together increased the half-life of DaunoXome^®^ between 4-5.6 h in comparison with free DNR ≈ 0.77 h [21,22]. The data shown indicate that DaunoXome^®^ has an improved pharmacokinetic profile in comparison with free DNR.

#### 3.1.3. Depocyt^®^

Depocyt^®^, a liposomal product containing cytarabine/Ara-C (Enzon Corporation, Piscataway, NJ, USA), was developed by SkyePharm Inc. (previously DepoTech Pharmaceuticals, La Jolla, CA, USA). Depocyt^®^ is a pyrogen-free, parenteral suspension of the antimetabolite Ara-C, developed for the treatment of neoplastic meningitis (NM) by controlled release of Ara-C. Depocyt^®^ is a slow-release formulation developed by encapsulating the aqueous drug solution in multivesicular particles with a granular structure known as DepoFoam™. DepoFoam™ technology consists of microscopic spherical particles (3–30 μm) and is suitable for encapsulating hydrophilic compounds such as Ara-C. These lipid foam-based particles are comprised of 96% aqueous foam and 4% biodegradable lipid [6]. The architecture of multivesicular DepoFoam™ particles provides a comparatively high drug-loading ability. They are bigger than standard unilamellar or multilamellar liposomes. The residues of lipid foam are biodegradable and metabolised by the usual metabolic pathways for triglycerides, phospholipids and cholesterol. Each vial contains 50 mg of Ara-C encapsulated in DepoFoam™ liposomes at a concentration of 10 mg/mL. Each 5-mL vial contains 50 mg Ara-C, 22 mg cholesterol, 6 mg triolein, 28.5 mg dioleoylphosphatidylcholine (DOPC) and 5 mg dipalmitoylphosphatidylglycerol (DPPG) suspended in 0.9% preservative-free saline. The Depocyt^®^ suspension is maintained at a final pH of 5.5 to 8.5. Due to the higher density of DepoFoam™ particles than that of the suspending medium, these particles have a tendency to settle at the bottom over time, and therefore the suspension require gentle agitation to re-suspend the particles before injection [6]. The recommended adult dosage of Ara-C is 50 mg (5 mL Depocyt^®^) once every two weeks.

A phase I/II clinical trials for ventricular and lumbar-administrated Depocyt^®^ pharmacokinetic studies was initiated, in which cytotoxic CSF Ara-C levels were maintained for more than 14 days in both the lumbar and ventricular fluid regardless of the site of drug administration [24]. The terminal half-life of intraventricular Depocyt^®^ was 141 h, whereas that of standard Ara-C was 3.4 h [25]. In another clinical study Depocyt^®^ was compared to methotrexate in patients with solid tumour neoplastic meningitis and results revealed similar response rates. Also, Depocyt^®^ significantly increased the phase to neurological progression and the effectiveness of Ara-C is a function of both the concentration and duration of exposure. Depocyt^®^ has the ability to destroy tumour cells more effectively in the meninges and CSF than standard Ara-C formulations [26].

#### 3.1.4. Myocet^®^

Myocet^®^ is a nonpegylated liposomal DOX that has been approved in combination with cyclophosphamide for first-line treatment of patients with breast cancer. Myocet^®^ (Elan Pharmaceuticals, Princeton, NJ, USA) was developed to reduce the cardiotoxicity of DOX while maintaining its anti-tumour efficacy [27]. Myocet^®^ liposomes are about 150 to 250 nm in size and contains cholesterol and the acidic egg phosphatidylcholine (EPC) in a molar ratio of 45:55. The drug to lipid ratio is about 0.27. The larger size of these liposomes makes them easily recognised by the mononuclear phagocyte system (MPS). The large size of vesicles also minimises their exposure to normal tissues, due to which some acute and chronic toxicities were diminished [28]. Active loading for amphipathic weak bases was employed for the development of Myocet^®^ liposomes. Blank liposomes were prepared in an acidic citrate buffer (pH 4.0, 300 mM citrate) followed by the addition of sodium carbonate to increase the pH outside these liposomes to approximately 7.3. Finally, these liposomes are incubated with DOX and slightly heated briefly. As a result, DOX goes across the lipid bilayer and becomes protonated inside the liposomal aqueous core. The negatively charged membrane-associated lipids serve to form “ion pairs” with DOX (which is positively charged at a physiological pH), which favours entry of DOX into the liposome [29]. Once DOX becomes protonated, it has trouble crossing the lipid bilayer, which results in entrapment efficiencies of more than 99% [30].

Myocet^®^ was compared with free DOX in preclinical toxicity studies performed on Beagle dogs, in which Myocet^®^ has shown a better toxicity profile than free DOX [28]. Harasym and co-workers reported the highest tumour concentrations were 2–3 times higher for Myocet^®^ as compared to free DOX in a solid tumour model and for the ascitic model the maximal level in tumour drug exposure was 10 times higher for Myocet^®^ as compared to free DOX [31]. These results encouraged the selection of Myocet^®^ for clinical studies. A phase I clinical study was performed on 38 patients with refractory solid tumours, in which Myocet^®^ and free DOX were given through i.v. injection at same dose. The study demonstrated reduced myelosuppression and gastrointestinal adverse effects due to Myocet^®^ as compared with free DOX [30]. In a phase III clinical study in patients with metastatic breast cancer, Myocet^®^ demonstrated similar response rates and progression-free survival times. Furthermore, the occurrence of cardiac events and congestive heart failure was significantly lower for Myocet^®^ [32]. Batist and co-workers [33] conducted another multicentric designed clinical trial in patients with metastatic breast cancer, in which a Myocet^®^ (60 mg/m^2^) and cyclophosphamide (600 mg/m^2^) combination was compared with a free DOX and cyclophosphamide combination at the same dose. The results showed equivalent efficacy with minimal toxicity due to a combination of Myocet^®^ with cyclophosphamide.

#### 3.1.5. Mepact^®^

Mepact^®^ is a mifamurtide (MFT) containing liposomes commercialised by Takeda Pharmaceutical Company Limited, previously IDM Pharma SAS. Mepact^®^ designated as an ‘orphan’ drug by the European Medicines Agency (EMA) in 2004 and it was the first drug approved for the management of high-grade, resectable, non-metastatic bone tumours combined with postoperative combination chemotherapy in children, adolescents and young adults who have gone through full macroscopic surgical resection. Mepact^®^ contains <100 nm multilamellar vesicles of liposome encapsulated muramyl tripeptide phosphatidylethanolamine (L-MTP-PE) [34]. MTP-PE is a fabricated lipophilic derivative of muramyl dipeptide (MDP) (a naturally occurring constituent of bacterial cell walls) and it is a conjugate of MTP and dipalmitoylphosphatidylethanolamine (DPPE). Muramyl dipeptide activates monocytes, macrophages and cytokines like tumour necrosis factor alpha, interleukin-1b, interleukin-6, interleukin-8 and interleukin-12. Synthetic lipids used in the preparation of Mepact^®^ liposomes, i.e., dioleoyl-phosphatidylserine (DOPS) and 1-palmitoyl-2-oleoyl-phosphatidylcholine (POPC), are at a 3:7 molar ratio (1g total lipid/vial) with MTP-PE (4 mg/vial) [35]. The anti-osteosarcoma effects produced by MFT in vivo are due to an immune response against osteosarcoma lung metastases, even though the drug showed no cytotoxicity towards normal or tumour cells in vitro. Macrophage cells possess a “flipped phosphatidyl serine” to the external membrane after apoptosis from chemotherapy; therefore, phosphatidyl serine containing lipids provides the signal to these cells and thus both mifamurtide’s active and inactive constituents target the immune cells of the lungs [36].

The pharmacological parameters of liposomal MFT were characterised in healthy adults and in patients with high-risk, metastatic and recurrent osteogenic sarcoma [37]. The results showed that single MFT at a single 4 mg dose can be safely given to healthy adult volunteers and that pharmacokinetic variability is less for MFT [the coefficient of variation (% CV) in both the AUC and the maximal concentration (*C*_max_) was less than 30%]. A phase III clinical trial of L-MTP-PE given in addition to the usual combination chemotherapy conducted in children and young adults with osteogenic sarcoma showed an increase in six-year net survival from 70% to 78% [38].

#### 3.1.6. Marqibo^®^

Marqibo^®^, vincristine (VCR) sulfate liposomal injection (VSLI), developed by Talon Therapeutics, Inc. USA, was approved for the treatment of adult patients with Philadelphia chromosome-negative (Ph^−^) acute lymphoblastic leukaemia (ALL) with second or greater relapse or whose disease has advanced after two or more anti-leukaemia therapies. Each vial contains 5 mg/31 mL (0.16 mg/mL) VCR sulphate for a single dose. VCR is encapsulated in an aqueous interior core of sphingomyelin/cholesterol liposome called optisomes [39]. These optisomes were specifically developed to promote the higher loading and holding of VCR. Also, these optisomes increase the circulation time of enclosed VCR and slowly release the drug into the tumour vasculature. These factors lead to enhanced activity due to the high concentration of encapsulated drug in target tissues. Sphingomyelin (SM) and cholesterol at a molar ratio of approximately 60:40 (mol:mol) are present in the VSLI, with an approximate liposome mean diameter of 100 nm. The SM/Cholesterol lipid constituent and the small mean particle diameter of the VSLI liposome contribute to low protein binding, which results in a prolonged circulation time for the liposome. More than 95% of the drug is encapsulated in the liposomes [40].

Marqibo^®^ has prolonged plasma circulation compared with free VCR and passively targets VCR to tumours by discharging through the fenestrations that characterise the tumour neovasculature [41]. A phase II clinical trial, single-arm open study in patients with relapsed or refractory aggressive non-Hodgkin lymphoma, analysed the efficacy and safety of Marqibo^®^ as a single agent. Marqibo^®^ was given at approximately twice the dose intensity of standard-free VCR. Patients in this study demonstrated a comparable toxicity profile to standard-free VCR [42].

#### 3.1.7. Onivyde™

Onivyde™, an irinotecan (IRI) liposome injection, is a product of Merrimack Pharmaceuticals Inc. approved in 2015. Onivyde™ coupled with leucovorin and fluorouracil is indicated for the management of patients with metastatic adenocarcinoma of the pancreas that showed disease progression after gemcitabine-based therapy. Onivyde™ is formulated with a water-soluble semisynthetic IRI hydrochloride trihydrate, a topoisomerase inhibitor, into a liposomal dispersion. Onivyde™ liposomes are unilamellar lipid bilayer vesicles with a mean diameter of 110 nm that encapsulates IRI in a gelated or precipitated state as the sucrose octasulphate salt using an ion-exchange/titration method in aqueous space. Onivyde™ was prepared by a novel method, i.e., intra-liposomal drug stabilization technology, which encapsulates drug into long circulating liposome-based nano-vesicles [43]. In this technology, polymeric or nonpolymeric highly charged anions and intra-liposomal trapping agents like polyphosphate or sucrose octasulfate were used. A high-p*K*a polyalkylamine gradient was utilised in this technology. This facilitates the encapsulation of IRI at a high drug:lipid ratio (more than 800 g IRI per mol of phospholipid) within the liposomes. The half-life of drug release in the system was also shown to be increased up to 56.8 h. The vesicle is composed of DSPC, cholesterol and methoxy-terminated polyethylene glycol (MW 2000)-distearoylphosphatidyl ethanolamine (MPEG-2000-DSPE) in the ratio of 3:2:0.015, which encapsulated more than 90% of the drug [44].

Liposomal IRI was compared with free IRI using human colon (HT29) and breast (BT474) cancer xenograft models. Liposomal IRI showed significantly enhanced cytotoxic activity due to exponentially higher drug loading and extended drug retention in vivo [43]. A randomised, open-label NAPOLI-1 clinical trial was conducted on patients with metastatic pancreatic adenocarcinoma whose cancer had progressed after consuming the chemotherapeutic agent gemcitabine or a gemcitabine-based therapy demonstrated efficacy and safety of Onivyde™. The patients in the study who consumed fluorouracil/leucovorin with Onivyde™ survived 6.1 months on average, compared with 4.2 months on average for patients who consumed either fluorouracil or leucovorin. In another study patients who consumed fluorouracil/leucovorin with Onivyde™ had an average delay of 3.1 months in the amount of time required for tumour progression compared with 1.5 months for those who consumed either fluorouracil or leucovorin [45].

### 3.2. Liposomes for Fungal Infections

#### 3.2.1. Abelcet^®^

Abelcet^®^, amphotericin B (AmB) lipid complex formulation, is a sterile, preservative-free suspension for i.v. infusion. Sigma-Tau Pharmaceuticals developed Abelcet^®^ in 1995 for the treatment of invasive fungal infections refractory to conventional AmB desoxycholate therapy or when renal impairment or unacceptable toxicity precludes use conventional AmB. Abelcet^®^ is a unique formulation, combined with two phospholipids in a 1:1 drug:lipid weight ratio; the stoichiometry of the molecular complex that forms between AmB and lipid is also nearly a 1:1 drug:lipid molar ratio. The lipid comprises of dimyristoyl phosphatidylcholine (DMPC) and dimyristoyl phosphatidylglycerol (DMPG) combined in a 7:3 molar ratio. The concentration of the drug in the complex is from about 25 to about 50 mol % [46]. Abelcet^®^, when seen in a freeze-fracture electron microscope, appeared as ribbon-like structures [47], consisting of stable drug–phospholipid single layers or rosettes of large and variable size (1 to 10 mm diameter) [48]. In spite of the high concentration of AmB in the Abelcet^®^, there is immediate release of AmB into the system following infusion. Pharmacokinetic studies demonstrate that there is deposition in the RES [49]. This “depot” form releases the drug at local sites of infection, may be through the action of lipase [50].

Multiple doses of Abelcet^®^ (5 mg/kg/day for five to seven days) vs. AmB deoxycholate (0.6 mg/kg/day for 42 days) were compared. Maximum plasma concentration of about 1.7 ± 0.8 vs. 1.1 ± 0.2 μg/mL, AUC 14 ± 7 vs. 17.1 ± 5 μg*h/mL, clearance 436 ± 188.5 vs. 38 ± 15 mL/h*kg, volume of distribution 131 ± 57.7 vs. 5 ± 2.8 L/kg and half-life 173.4 ± 78 vs. 91.1 ± 40.9 h were reported for Abelcet^®^ and AmB deoxycholate, respectively. The high clearance and large volume of distribution values from the blood of AmB following the administration of Abelcet^®^ possibly indicate uptake by tissues. The long terminal elimination half-life apparently indicates a slow redistribution from tissues. Even though AmB is cleared slowly, there is a low concentration in the blood after multiple dosing [51].

#### 3.2.2. Ambisome^®^

Ambisome^®^ liposome for injection is a sterile, non-pyrogenic freeze-dried product for i.v. infusion. It is a product of Astellas Pharma USA approved in 1997. Ambisome^®^ is approved for the treatment of serious, life-threatening fungal infections including leishmaniasis, aspergillosis, blastomycosis, coccidioidomycosis in febrile, neutropenic patients and a certain form of meningitis in people infected with HIV. Ambisome^®^ is also prescribed for the treatment of invasive systemic infections caused by *Aspergillus, Candida*, or *Cryptococcus* in patients those cannot tolerate conventional AmB therapy or renally impaired patients.

In Ambisome^®^, charged complexes were observed between positively charged mycosamine of AmB and the negatively charged distearoylphosphatidylglycerol (DSPG). It also showed hydrophobic interactions with cholesterol constituents of the lipid membrane, due to which AmB gets incorporated tightly within the liposomal membrane. The lipid bilayer of Ambisome^®^ is composed of hydrogenated soy phosphatidylcholine, cholesterol, DSPG and AmB in a 2:1:0.8:0.4 molar ratio [52]. The stability of the liposomal membrane in Ambisome^®^ is achieved by employing saturated (rigid) phospholipids and a charged phospholipid (phosphatidylglycerol) in combination with cholesterol. Interaction between AmB with the cholesterol is direct and occurs via its sterol binding region, which also plays a role in stabilization. Ambisome^®^ liposomes small size (100 nm) provides prolonged circulation in the plasma, along with in vivo stabilization. The content of Ambisome^®^ was kept low, i.e., about 10% by weight, which helps in maintaining a ‘true’ liposomal formulation [53].

Preclinical data reports showed negligible haemolysis caused by Ambisome^®^ [53]. Ambisome^®^ demonstrated increased safety in animal models with systemic fungal infection. It was tolerated at doses higher than those of conventional AmB and possessed a higher therapeutic index [52]. Ambisome^®^ was also shown to retain the pharmacological properties of AmB for a broad range of fungi, including *Candida*, *Cryptococcus*, *Aspergillus*, *Blastomyces* and *Paracoccidioides* in various preclinical studies [52]. Although Ambisome^®^ has a different plasma pharmacokinetic profile than lipid complex formulations [54], it remains in the plasma compartment for a longer duration, resulting in higher plasma levels compared to conventional AmB. On the other hand, the lipid complexes get rapidly cleared. Ambisome^®^ still accumulates mainly in MPS-related tissues like the liver and spleen in spite of its slow clearance [55].

#### 3.2.3. Amphotec^®^

Amphotec^®^, AmB cholesteryl sulphate complex for injection is a parenteral freeze dried lipid-based formulation to receive US FDA approval. Ben Venue Laboratories Inc., Bedford, OH, USA developed Amphotec^®^ in 1996 for the treatment of serious fungal infections and leishmaniasis in patients where renal disease or higher toxicity prevents the use of AmB in efficacious doses and in patients with systemic aspergillosis where previous AmB deoxycholate therapy has failed. Guo et al. first prepared a novel drug dosage form based on the particular interaction of AmB with sterols. The sodium salt of cholesteryl sulphate (CS), a naturally existing metabolite of cholesterol, forms a thermodynamically stable colloidal complex with AmB at a 1:1 drug to lipid molar ratio. Amphotec^®^ is composed totally of uniform small spherical unilamellar lipid vesicles less than 100 nm in size [56]. Amphotec^®^ forms a colloidal suspension of microscopic disc-shaped particles when reconstituted in an aqueous solvent. The rigidity of the lipid components offers stability to the bilayer structures [56]. When compared with a conventional AmB formulation, this unique colloidal formulation of AmB reduces haemolysis, acute toxicity and lipoprotein binding [56]. It may be that the reduction of renal toxicity is because of the strong interaction of AmB with the cholesteryl sulphate of the formulation, which decreases the amount of free AmB in circulation.

The increased safety profile of Amphotec^®^ (above conventional AmB) was demonstrated in repeated animal studies, which showed a 5–8-fold reduction in renal toxicity as compared to conventional AmB. Amphotec^®^ gets eliminated quickly from the circulation, mainly from MPS tissues, where it accumulates in a comparatively non-toxic form [57,58]. In a Phase I clinical trial, Amphotec^®^ was shown to be safe at doses higher than conventional AmB, with an identical pattern of acute adverse effects with decreased renal toxicity [59]. Amphotec^®^ at different doses of up to 8 mg/kg/day exhibited high response rates against fungal infections like candidiasis, aspergillosis and coccidioidomycosis in patients who were unresponsive or hypersensitive to conventional AmB treatment in various clinical trials [60,61]. Therefore, Amphotec^®^ was significantly less nephrotoxic than conventional AmB and could be given to patients with renal diseases.

### 3.3. Liposomes for Photodynamic Therapy

#### Visudyne^®^

Visudyne^®^, a product of Novartis AG, Switzerland, is the first light-activated drug available for the treatment of patients with predominantly classic subfoveal choroidal neovascularization due to age-related macular degeneration (AMD). Visudyne^®^ was designed to eradicate the abnormal blood vessels in the eye related with conditions such as the wet form of macular degeneration. Visudyne^®^ contains verteporfin (VPF), a synthetic chlorine-like porphyrin, which has a light absorption peak at 692 nm and is utilised as a photosensitiser for photodynamic therapy (PDT). Photodynamic therapy may offer selective eradication of the neovascular membrane while producing minimal damage to retinal and choroidal tissues. This treatment modality uses low-intensity light at a wavelength within the absorption band of the injected dye to irradiate photosensitised tissues and cause local cytotoxic effects by photochemical reactions. Visudyne^®^ liposome is a unilamellar phospholipid vesicle based on lipids DMPC and egg phosphatidyl glycerol (EPG). The molar ratio of photosensitiser and mixture of phospholipids is about 1:8.0 and the size of the liposomes is between 150 and 300 nm [62]. The lipophilicity of VPF resulted in 100% efficiency of incorporation into the liposome. In addition, it is readily reconstituted to a stable liquid form and results in accurate concentration.

Biodistribution studies for liposomal VPF and aqueous VPF showed slightly higher accumulation of VPF in tumour tissue with liposomal VPF than with aqueous VPF. Clearance rates were found to be almost equivalent, i.e., half-lives of 16.1 h for liposomal VPF and 16.9 h for aqueous VPF. In a bioassay in tumour-bearing mice, when PDT was administered 3 h after i.v. administration of liposomal VPF and aqueous VPF preparation, the former was superior to the aqueous VPF preparation. In vitro plasma distribution studies demonstrated the distribution of liposomal VPF and free VPF as 91.1% ± 0.3% and 49.1% ± 2.6%, respectively. [63]. Age-related macular degeneration with PDT analysis was the subject of two phase III clinical studies, where liposomal VPF was compared with placebo. Photodynamic therapy with VPF liposomes in selected patients with neovascular AMD lessened the possibility of moderate and severe vision loss. These advantages were retained for at least 24 months of follow-up [64].

### 3.4. Liposomes for Pain Management

#### 3.4.1. DepoDur™

Epidural morphine sulphate sustained-release liposome injection DepoDur™ is a novel drug of SkyePharma, San Diego, CA, approved in 2004. DepoDur™ is intended for administration before surgery or following clamping of the umbilical cord during a caesarean section. It has been employed as a single-dose administration at the lumbar level by the epidural route.

DepoDur™ is composed of multivesicular lipid-based particles with median diameter in the range of 17 to 23 μm. Each vial contains 10 mg/mL of morphine sulphate encapsulated into liposomes that are further dispersed in a 0.9% sodium chloride preservative-free suspension. Phospholipids and other ingredients are cholesterol, 3.3 mg/mL; DOPC, 4.2 mg/mL; DPPG, 0.9 mg/mL; tricaprylin, 0.3 mg/mL; and triolein, 0.1 mg/mL [65]. In DepoDur™ formulation, morphine is encapsulated in lipid foam by DepoFoam™ Technology. The lipid foam encapsulation of morphine permits sustained release of morphine into the epidural space for a longer time. This foam is characterised by a specific multivesicular structure of micron-size, nonconcentric aqueous chambers that encapsulate the active drug. It ruptures in a time-dependent fashion to release the encapsulated drug into the epidural space and the constituents of the foam was designed to release the morphine for a 48-h period [66].

In preclinical studies, Kim et al. observed that the release of morphine sulphate was sustained from DepoFoam™. These liposomes were given epidurally in rats and compared with systemically given morphine sulphate. Results showed that a single dose of sustained-release morphine sulphate through the epidural route was sufficient to achieve a similar onset time of maximum analgesia. It also provides significantly prolonged analgesia compared to systemic morphine sulphate. The maximal concentration for sustained-release morphine was 6% in serum and 32% in cerebrospinal fluid (CSF) compared with systemic morphine sulphate. Furthermore, a 30-fold increase in the CSF terminal half-life was detected for sustained-release morphine [67]. A pilot study was conducted in which a single dose of extended-release epidural morphine (EREM) could provide safe and effective post-operative pain relief after total hip arthroplasty under spinal anaesthesia [68]. Patients received EREM 10, 20 or 30 mg and the other group received conventional epidural morphine. The use of EREM results in decreased total fentanyl requirements, longer time to rescue and lower pain ratings over 48 h of study.

#### 3.4.2. Exparel^®^

Exparel^®^, a bupivacaine extended-release liposome injection, is a new liposomal local anaesthetic formulation. Pacira Pharmaceuticals, Inc. has developed Exparel^®^, approved by the U.S. FDA in 2011. Exparel^®^ is designed for extended release of the drug up to 72 h. Exparel^®^ is a multivesicular liposomal formulation of bupivacaine based on Depofoam™ technology, being developed for postsurgical analgesia [69]. Exparel^®^ contains a novel phospholipid excipient, dierucoylphosphatidylcholine (DEPC), which is unique to this product and has not previously been included in other DepoFoam™-based approved products, e.g. DepoDur™ and DepoCyt^®^ [70]. Other lipid components present in Exparel^®^ are cholesterol, DPPG and tricaprylin. The median diameter of the liposome particles ranges from 24 to 31 μm [70].

Subcutaneous (s.c.) administration of 20 mL of 2% bupivacaine liposomes vs. 20 mL of 0.5% plain bupivacaine in human volunteers was compared by Davidson et al. [71]. The *C*_max_ values found between the two groups i.e., vs. 0.83 ± 0.34 vs. 0.87 ± 0.45 µg/mL in liposomal and plain groups, respectively, show no difference in spite of a 4-fold higher bupivacaine dose. The terminal half-life increases by 9.8-fold in the liposomal bupivacaine group i.e., 1294 ± 860 vs. 131 ± 58 min. A phase II multi-centre clinical trial confirmed the 7-fold increase in *t*_max_ in the liposomal bupivacaine group compared with the plain bupivacaine group, which was attributed to the sustained release of liposomal bupivacaine [72].

### 3.5. Liposomes for Viral Infections

#### 3.5.1. Epaxal^®^

Epaxal^®^ is the first virosome-adjuvanted vaccine for hepatitis A (HAV). It was the first product based on the liposomal vaccine or virosome technology developed and patented by Crucell Berna Biotech, Switzerland. Epaxal^®^ has a high tolerability and the absence of aluminium and thiomersal in this vaccine allows for intradural or s.c. administration since it causes fewer adverse local effects compared with conventional aluminium-adsorbed vaccines. Epaxal^®^ is highly effective after administration of the first dose, offering protective immunity for a limited duration. It provides immunity for up to 20 years following the second booster dose [73]. In contrast to liposomes, virosomes contain viral envelope glycoproteins intercalated in the phospholipid bilayer membrane. The HAV vaccine Epaxal^®^ is based on formalin-deactivated RG-SB strain HAV particles, which are combined to the surface of virosomes. Virosomes are spherical vesicles composed of phospholipids, lecithin, phosphatidylcholine and phosphatidylethanolamine. They form unilamellar vesicles of approximately 150 nm in diameter. The lipid components of Epaxal^®^ virosomes are 1,2-Dioleoyl-sn-glycero-3-phosphoethanolamine (DOPE) and DOPC present in a molar ratio of 25:75 [74]. The RG-SB HAV strain was produced on an MRC-5 human diploid cell culture and then the virus was purified from disrupted cells by ultrafiltration. HAV was deactivated by treatment with formalin and then attached to the virosome surface. This structure facilitates the delivery of the HAV antigen to immunocompetent cells owing to the properties of fusion-active glycoproteins [75]. A well-designed clinical trial study on adults [76] and children aged 1.5 to 6 years [77] demonstrated the efficacy, tolerability and immunogenicity of Epaxal^®^. After a booster vaccination with Epaxal^®^ in adults, more than 95% of subjects were protected from HAV infection for more than 20 years [78]. In general, details on pharmacokinetic properties of vaccines have not been extensively reported.

#### 3.5.2. Inflexal^®^ V

Inflexal^®^ V, an inactivated, virosomal-adjuvanted influenza vaccine, was developed and patented by the Crucell Berna Biotech, Switzerland. Inflexal^®^ V is composed of haemagglutinin surface molecules of the influenza viruses, which are kept on the double membrane of lecithin-phospholipid liposomes. Inflexal^®^ V contains 15 µg haemagglutinin each of influenza A and B virus strains. Inflexal^®^ V virosomes are prepared by a combination of natural and synthetic phospholipids after the removal of influenza surface glycoproteins, neuraminidase (NA) and hemagglutinin (HA) by detergent treatment. The phospholipids of Inflexal^®^ V virosomes are composed of 70% lecithin and 20% cephalin as structural components. Cephalin additionally stimulates B cells independently of T-cell determinants and can bind hepatitis A antigen. Inflexal^®^ V contains 10% envelope phospholipids in the molar ratio of 75:25 DOPC:DOPE [79]. The resulting virosomes are spherical, unilamellar vesicles with approximately 150 nm mean diameter. Due to the low viral and avian protein content, they are almost non-immunogenic phospholipids vesicles. The superior immunogenicity of Inflexal^®^ V vs. conventional influenza vaccines has shown statistically significant improvement above other vaccines [80]. The superior immunogenicity and tolerability of Inflexal^®^ V has also been shown by a comparison with Influvac, a subunit vaccine [81].

## 4. Liposomal Formulations in Clinical Trials

**Figure 6:**
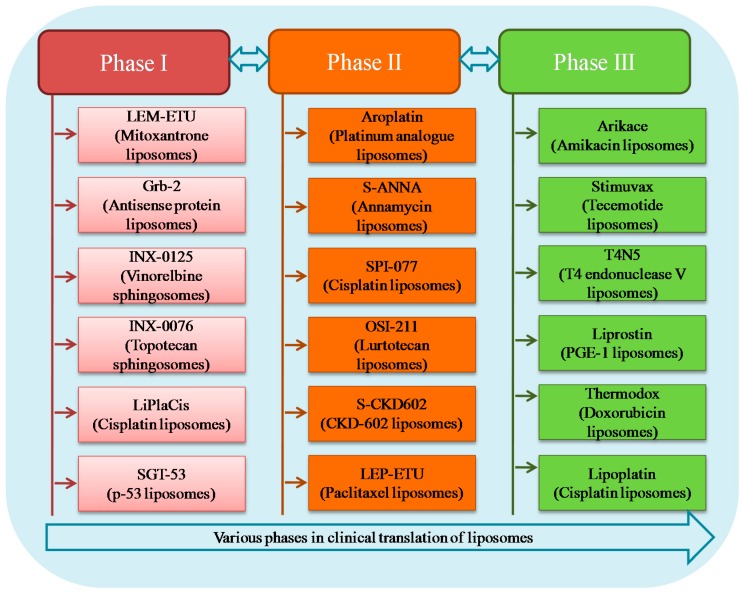
Liposomes present under different phases of clinical trial investigation.

Intensive research on lipid carriers has led to the development of many new liposomal formulations for the management of various ailments. Figure 6 represents various products undergoing clinical trial investigation along with their current development phase and indication. Table 2 shows details of different liposomal products in terms of liposome composition, indication and route of administration.

### 4.1. Phase III

#### 4.1.1. Arikace™

Arikace™ (Transave, Inc.,) is a prolonged release amikacin (AMK) liposomal formulation for inhalation, developed using advanced pulmonary liposome technology. AMK is classified as belonging to the aminoglycoside class antibiotics, which act by inhibiting the production of bacterial proteins. It is indicated in the treatment of certain serious bacterial infections [82]. Transave, Inc. developed Arikace™ for site-specific treatment of serious lung infections. Arikace™ received orphan drug status from the FDA in the United States and the European Medicines Agency in Europe for the treatment of *Pseudomonas aeruginosa* (PsA) infections in patients with Cystic Fibrosis (CF). Product also received orphan drug status by FDA for *Pseudomonas*-associated non-CF bronchiectasis therapy. Transave Inc. got merged with Insmed Inc. in 2010 and the combined company filed for its orphan status with FDA and European Medicines Agency in 2011 for lung infections due to non-TB Mycobacteria (NTM). The product at present is in phase III clinical trials for these indications [83].

AMK is entrapped into liposomes (0.2–0.3 µm) composed of neutral, biocompatible lipids, i.e., DPPC and cholesterol. Arikace™ has been administered once daily at different doses ranging from 70 to 560 via an electronic nebuliser (eFlow^®^). This device ensures the delivery of small droplet sizes (1–5 µ) to facilitate more efficient distribution in the lungs. The formulation enables the penetration of the drug into biofilm and sustains the release of AMK in the lungs with minimal systemic exposure. The liposomal formulation achieved high lung *C*_max_, AUC, *t*_1/2_ and improved AUC:MIC ratio. It resulted in the potential inhibition of PsA pathogens, including its resistant isolates. Moreover, virulence factors secreted by PsA facilitated the further release of AMK from Arikace™ [84].

Toxicology studies in dogs and rats performed for a period of 3–6 months supported long-term clinical studies. Phase II blinded and placebo-controlled as well as multi-cycle open-label studies were performed in CF patients with chronic PsA infection for 28 days on and 56 days off cycle with once a day administration of the drug. Changes in Pulmonary Function Testing (PFT), Forced Expiratory Volume (FEV) and Colony-Forming Units (CFU) were considered as end-points of the study. Positive results were obtained from phase II studies, where there was a clinically significant improvement in lung function at the end of treatment. The observed clinical effect was dose-dependent, whereby the group that received a 560-mg dose showed a significant increase in FEV of 17.6% compared to the placebo. The product was well tolerated and no considerable difference was observed in adverse events between the Arikace™-treated and placebo groups. In summary, once a day administration of Arikace™ in CF patients with PsA infections demonstrated safety, tolerability, biologic activity and efficacy [85]. Similarly, in a randomised multi-centre study of Arikace™ in patients with NTM lung infections, a change from baseline on the semi-quantitative scale and NTM culture conversion to negative were taken as end-points. Liposomal AMK showed statistical significance in culture conversion compared to the placebo. The data obtained from clinical trials of Arikace™ confirmed the magnitude and sustained improvement of lung function. Arikace™ may be an advancement in terms of treatment options for CF patients with PsA lung infections as well as patients with NTM lung infections [86].

#### 4.1.2. Stimuvax^®^

Stimuvax^®^, earlier known by the name BLP25 liposome vaccine, is a therapeutic vaccine indicated for certain types of cancer expressing Tumour-Specific Antigens (TSA). This cancer vaccine incorporates an antigenic lipo-peptide, i.e., Tecemotide (TCM), in a liposomal delivery system. TCM targets mucin 1 (MUC1), which is overexpressed in different cancer cells, including breast, prostate, non-small cell lung cancer (NSCLC) and colorectal cancer. MUC1 gets abnormally glycosylated when a cell becomes cancerous in nature. Stimuvax, upon targeting TSA, triggers a cellular immune reaction which leads to immune rejection of tumours that have the MUC1 antigen. Although it is referred to as a cancer vaccine, it does not actually prevent cancer but aids in enhancing the life expectancy of cancer patients. The product was initially developed by Cancer Research UK and was taken forward by Oncothyreon, Inc. (formerly known as Biomira, Inc.) [87]. In 2008, Merck KgaA acquired the product rights to Oncothyreon. Stimuvax^®^ showed a positive response, i.e., a significant increase in life expectancy from 13.3 to 30.6 months, in its phase II trials. As a shocking disclosure in the field of cancer research, Stimuvax^®^ faced failure in phase III trials as the vaccine did not meet its primary or secondary end-points of the study, which led to its termination [88].

The liposomal vaccine is composed of synthetic MUC1 lipopeptide (antigen), monophosphoryl lipid A (immunoadjuvant), cholesterol, DMPG and DPPC lipids. This vaccine is a lyophilised powder, which contains 300 μg of TCM and 150 μg of monophosphoryl lipid A per vial. Patients are treated with a 300 mg/m^2^ cyclophosphamide i.v. dose followed by weekly s.c. injections of Stimuvax^®^ for eight consecutive weeks in a phase II study. Vaccination with Stimuvax^®^ stimulates immune-response-mediated, MUC1-specific cytotoxic T lymphocytes (CTL), which destroys cancer cells [89,90].

A positive outcome was obtained in stage III and IV NSCLC patients in an early-stage trial with Stimuvax^®^. It was the first product belonging to the class of cancer vaccines that entered advanced clinical trial phase III. Three worldwide phase III clinical trials, namely START (Stimulating Targeted Antigenic Responses to NSCLC), INSPIRE (Stimuvax^®^ trial In Asian NSCLC Patients: Stimulating Immune REsponse) and STRIDE (STimulating immune Response In aDvanced brEast cancer), were initiated and sponsored by EMD Serono for Stimuvax^®^. Although the START study failed to meet its primary end-point, there was significant survival advantage in the subgroup of patients, which led to the START 2 and INSPIRE trials [91]. However, the product failed to meet its primary or secondary end-points, which led to the unfortunate termination of the Stimuvax^®^ trial. In 2014, the company announced that it was discontinuing its worldwide clinical trials associated with TCM [88].

#### 4.1.3. T4N5

T4N5 liposomal lotion, also known as bacteriophage T4 endonuclease V in liposomal lotion or Dimericine (DMC), has been developed to deliver DNA repair enzyme topically in Xeroderma pigmentosum patients [92]. This is a genetic disease making the individual susceptible to skin cancer. The liposomal lotion has also been reported to reduce the incidence of pre-malignant actinic keratosis by 68% and basal cell carcinoma by 30%. It is a lead product of AGI Dermatics, Inc., and the product received fast track designation in 2007 for Xeroderma pigmentosum in the USA. Upon administration of T4N5 lotion, T4-bacteriophage endonuclease V, a DNA repair enzyme, makes its entry into dermal cells. T4N5 then enters cell nuclei, where it attaches and incises pyrimidine dimers, which leads to catalysis of the first step of the cellular excision repair pathway. This further prevents pyrimidine dimers that inhibit DNA replication, which are produced within duplex DNA upon ultraviolet (UV) exposure. In vitro and in vivo studies showed that DMC improves the repair of UV-irradiation-associated DNA [92,93].

T4N5 liposomes are composed of egg lecithin and these are known to act by two different mechanisms, i.e., via removal of cyclobutane pyrimidine type DNA dimers or by restoring p53 gene function [94]. Phase I and phase II trials of DMC indicated for the prevention of skin cancer in Xeroderma pigmentosum patients were completed. However, phase III trials of this product were terminated in 2009. Although it seemed to be promising until phase II, the lack of expected clinical outcomes led to the termination of the T4N5 liposomes trial [95].

#### 4.1.4. Liprostin™

Liprostin™, or prostaglandin E-1 (PGE-1)-encapsulated liposomes, has been developed for the therapy of various cardiovascular diseases such as restenosis subsequent to angioplasty. Restenosis is associated with re-blockage of blood vessels in heart and legs following catheter intervention, which is a very expensive medical issue. PGE-1 is known to act as a potent vasodilator, platelet inhibitor, anti-inflammatory and anti-thrombotic agent [96]. Liprostin™ has been developed by Endovasc Ltd., a biopharmaceutical company focused on developing liposomes for generic products that have safety and efficacy. The company has three patents covering the use of Liprostin™ in various cardiovascular ailments. Liprostin™ improved the drug dynamics and improved the therapeutic index of various ailments including occlusive disease, limb salvage, claudication and arthritis [97]. Endovasc has been granted a conditional waiver by the FDA for clinical trials. AngioSoma acquired Liprostin™ in 2016 and phase II trials of the product were completed with excellent outcomes in peripheral vascular disorders, i.e., intermittent claudication. The company is planning a phase III trial for the product in treatment of intermittent claudication. This is the first clinical research trial where a vasoactive hormone like PGE1 has been used as an adjunct treatment along with angioplasty procedure [98]. The development of this product will have a significant, positive impact in terms of symptomatic relief by preventing blood vessel re-blockage.

#### 4.1.5. ThermoDox^®^

ThermoDox^®^, the only responsive liposomal formulation in clinical trials, is the temperature-sensitive liposomal formulation of approved drug DOX [99]. ThermoDox^®^ has been developed by Celsion Corporation, a leading oncology firm focused on the development of innovative anti-cancer drugs. It is indicated in primary liver cancer (hepatocellular carcinoma) and also recurring chest wall breast cancer. These liposomes are composed of DPPC, Myristoylstearoyl phosphatidylcholine (MSPC) and 1,2-distearoyl-sn-glycero-3-phosphoethanolamine-*N*-[amino(polyethylene glycol)-2000] (DSPE-PEG-2000). Three component lipids have been combined to attain the specific function and specific material properties associated with lipids, i.e., sharp thermal transition and rapid onset of membrane permeability [99]. This specific combination leads to the development of proprietary heat-activated DOX liposomes. The phase transition temperature of DPPC is 41.5 °C, and it undergoes phase change at 42 °C, which can be attained clinically by local hyperthermia. The addition of MSPC to the composition accelerates drug release by a slight reduction in the transition temperature of DPPC, while DSPE-PEG-2000 enhances the circulation time of liposomes. The presence of PEG lipid also helps in attaining lysolipid-induced permeability at a faster rate. The potential of stimulating drug release in tumour targeting has been realised with ThermoDox^®^ [100,101].

ThermoDox^®^ is administered by the i.v. route in combination with Radio-Frequency Ablation (RFA) [102]. This product can also be used in combination with microwave hyperthermia or high-intensity focused ultrasound [103]. Local hyperthermia (39.5–42 °C) induced by RFA release the encapsulated DOX from liposomes and attains high drug concentrations in the targeted tumour. ThermoDox^®^ achieved 25-fold higher drug concentration in the target site than a normal i.v. infusion and 5-fold higher drug concentration compared to standard DOX liposomes. Enhanced release of DOX from liposomes is due to grain boundary permeabilisation at the phase transition temperature [104]. This product received orphan drug designation from the European Commission, USA orphan drug status and FDA fast track designation for the treatment of hepatocellular carcinoma. Currently, phase III trials (HEAT study) are being evaluated in patients with primary liver cancer, which is non-resectable. A phase III heat study has received regulatory agency support in 11 countries worldwide. Other clinical trials associated with the product are OPTIMA (treatment of hepatocellular carcinoma with ThermoDox with standardised RFA) and TARDOX (targeted chemotherapy using focused ultrasound for liver tumours) [105,106].

#### 4.1.6. Lipoplatin™

Lipoplatin™ is a proprietary liposomal formulation of Cisplatin (CPT), an FDA-approved, commercially available cytotoxic agent. CPT has been categorised under DNA cross-linking agents or DNA synthesis inhibitors. The product has been introduced as Lipoplatin™ for the treatment of pancreatic cancer and Nanoplatin™ for lung cancer. This product has been developed by Regulon Inc., an oncology-focused drug delivery firm aimed at developing improved versions of leading chemotherapy drugs [107]. Lipoplatin™ is composed of lipids including DPPG, soy PC, MPEG-DSPE lipid conjugate and cholesterol. CPT liposomes have been indicated against various indications, i.e., pancreatic cancer, non-small cell lung cancer, testicular and ovarian carcinoma, head and neck cancer as well as bladder cancer. Encapsulation of CPT into liposomes offer various advantages in terms of high encapsulation efficiency, long-term circulation in vivo, ability to attain 200-fold higher concentration in tumours compared to CPT alone and ability to penetrate the cell membrane [108].

Lipoplatin™ has been used in combination with gemcitabine in pancreatic cancer [109], while it has been used in combination with pemetrexed in NSCL. The product is presently in phase I trials for malignant pleural effusion, phase II trials for breast cancer and gastric cancer, phase II/III trials for pancreatic cancer and phase III trials for NSCL [110]. Lipoplatin™ has considerably reduced the adverse effects associated with CPT including renal toxicity, peripheral neuropathy, ototoxicity and myelotoxicity [111]. The European Medicines Agency granted orphan drug status to this product for pancreatic cancer treatment.

### 4.2. Phase II

#### 4.2.1. Aroplatin™

Aroplatin™ (L-NDDP, AR 726) is a chemotherapeutic platinum analogue *cis*-(*trans*-R,R-1,2-diaminocyclohexane) bis (neodecanoato) platinum (II) (NDDP) encapsulated liposomal product. This is the first liposomal platinum formulation to enter into clinical trials and the loaded analogue is structurally similar to Eloxatin (Oxaliplatin; Sanofi Aventis). These platinum analogues exhibit tumour cell cytotoxicity by forming inter- and intra-strand cross-links of DNA, thereby inhibiting its synthesis [112,113]. DMPC- and DMPG-composed multilamellar liposomes were prepared to encapsulate NDDP analogues. The liposomal formulation accumulates NDDP to a great extent in target sites when compared to CPT. The analogue, when delivered by L-NDDP, inhibits the emergence of liver metastasis of reticulosarcoma in mice, proving the formulation to be beneficial compared to the drug alone. The improved activity of liposomal formulation has been attributed to intraliposomal activation by reaction with DMPG. Liposomal formulation improved the bioavailability of NDDP and reduced its toxicity profile [114].

L-NDDP has been developed by Agenus Inc., while Aronex Pharmaceuticals procured the ownership for investigational new drug application of Aroplatin™. The product received orphan drug designation for malignant mesothelioma from the U.S. FDA. In 2002, a phase II monotherapy trial was completed for the product towards indication of metastatic colorectal cancer [115]. In 2002, a phase I/II trial of Aroplatin™ was completed for advanced solid malignancies. However, in 2005, after a phase I dose escalation trial of the product, as the maximum tolerated dose had been reached, the trial was closed and no further internal development of the product has been pursued. Besides the results observed from clinical trials, the imprecise chemical composition along with the instability of the drug in liposomes led to the termination of its clinical investigation. Almost 50% degradation of the complex was observed after reconstitution, where neodecanoic acid gets hydrolysed, which results in the further inactivation of drug [116].

#### 4.2.2. Liposomal Annamycin

Liposomal annamycin (L-Annamycin, Annamycin-LF, S-ANNA) is a liposomal formulation encapsulating the semi-synthetic DOX analogue annamycin. Liposomal vesicles of annamycin are composed of DMPC and DMPG in a 7:3 molar ratio. This liposomal composition allowed higher entrapment of the drug as well as flexibility in the selection of formulation, which delivers a higher proportion of drug to tumour [117]. This therapeutic moiety acts by DNA intercalation and the inhibition of topoisomerase II, further inhibiting DNA replication and protein synthesis. This formulation is also found to circumvent multidrug-resistance transporters, including p-glycoprotein. L-Annamycin has been developed to avoid the cardiotoxicity associated with available anthracyclines and is found to be less toxic with improved anti-tumour activity. Unmet medical need in the areas of acute myeloid leukaemia or acute lymphoid leukaemia necessitate the need for this product development [118,119]. This product has been developed by Aronex Pharmaceuticals and in 2015, Moleculin Biotech, Inc. made an agreement to acquire the rights for clinical stage development of L-Annamycin.

The product completed phase I and phase II trials in patients with refractory or relapsed acute lymphocytic leukaemia [120]. Both trials showed fewer dose-limiting toxicities compared to DOX and also improvement with respect to the clearance of leukaemic blasts [121]. Moleculin Biotech, Inc. has been planning accelerated approval of the product for acute myeloid leukaemia. The company also intends to apply for its orphan drug status in the USA for acute myeloid leukaemia.

#### 4.2.3. SPI-077

Liposomal CPT (SPI-077) is a formulation that encapsulates CPT within liposomal vesicles composed of a 51:44:5 molar ratio of hydrogenated soybean PC, cholesterol and *N*-(carbamoyl-methoxypolyethylene glycol 2000)-1, 2-distearoyl-sn-glycero-3-phospho-ethanolamine sodium salt [10]. The formulation contains 14 µg of CPT/mg of lipid in 110-nm liposomal vesicles. The clinical efficacy of CPT is limited by intrinsic resistance and systemic toxicity. STEALTH^®^, a (sterically stabilised) liposomal formulation of CPT (SPI-077), showed significant improvement in preclinical models, with appreciably greater tumour growth delay than CPT [122]. This is the first CPT liposomal formulation that entered clinical stage development. The product has been developed by Alza Corporation for the treatment of lung, head and neck cancer. The product is currently in phase II clinical trials, while pre-clinical trials showed that SPI-077 exhibited improvement of stability, prolonged circulation time, enhanced anti-tumour effect and decreased side effects compared with the free drug [123]. However, in clinical trials, the formulation failed to demonstrate efficacy, which may be due to incomplete release of the drug from liposomes at the target site.

#### 4.2.4. OSI-211

OSI-211 is a next-generation cytotoxic and liposomal formulation of lurtotecan (LRT) (OSI-211, NX 211), a novel topoisomerase I inhibitor that has applications in ovarian, head and neck cancer. The liposomes are composed of a 2:1 molar ratio of HSPC and cholesterol and the product is currently in phase II clinical trials. OSI Pharmaceuticals developed liposomes of LRT, which is another water-soluble camptothecin analogue [96]. The product demonstrated a 9%–67% increased drug accumulation in tumour accumulation in comparison to a drug delivered in 5% dextrose in preclinical trials. This product is being developed with the goal of developing a competitor to topotecan with superior efficacy and an equivalent or better toxicity profile for refractory ovarian cancer. OSI-211 showed a similar toxicity profile in a randomised phase II clinical trial when compared with topotecan in relapsed ovarian cancer treatment. The study of this formulation is ongoing in alternate patient populations or in alternate schedules [124].

#### 4.2.5. S-CKD602

S-CKD602is a pegylated liposomal formulation encapsulated with potent topoisomerase I inhibitor (CKD-602), developed by the Alza Corporation. This formulation has been associated with a long circulation property that assists in the improvement of AUC. CKD-602 is a semi-synthetic analogue of camptothecin, which, when encapsulated into liposomes, increased the AUC 50-fold compared to non-liposomal CKD-602 [125]. The STEALTH liposomes of the camptothecin analogue (CKD-602) are composed of a lipid bilayer with MPEG linked phospholipids on the outer surface. The inclusion of MPEG lipids into liposomes imparts properties of prolonged plasma circulation and improved drug delivery into tumours compared to conventional liposomes [113]. In preclinical studies conducted with these liposomes, there is a 3–10-fold increase in therapeutic index compared to the non-liposomal formulation. S-CKD602, presently under phase II trial investigation, exhibited an interesting property of greater distribution in fat compared to muscle tissue, which varies according to the body composition of a patient [126].

#### 4.2.6. LE-SN38

LE-SN38 is a liposomal product encapsulated with SN-38, which is an IRI active metabolite (Camptostar^®^, Pfizer, Inc.) that has been developed by NeoPharm Labs Ltd. [127]. Camptostar^®^ is a chemotherapeutic prodrug indicated for advanced colorectal cancer [128]. LE-SN38 is formulated by using NeoLipid^®^ patented technology with the goal of delivering the active drug without the need for conversion, which further minimises the variability and optimises the dose with minimum side effects. It is composed of a 50:40:10 molar ratio of DOPC, cholesterol and cardiolipin and a drug to lipid ratio of 1:18 [127]. LE-SN38 improved the pharmacodynamic profile of a relatively insoluble compound, SN38, in parallel with an improved safety and efficacy profile when compared with the pro-drug version. The product demonstrated safety and tolerability in its phase I trial investigation. SN-38, when delivered via liposomes, achieved similar blood levels and a systemic effect comparable to or greater than Camptostar^®^. The product is currently undergoing phase II clinical trial investigation for patients with metastatic colorectal cancer [129].

#### 4.2.7. LEP-ETU 

LEP-ETU is a liposomal formulation of paclitaxel (PTX); ETU in the code represents an easy-to-use formulation. PTX is a well-established therapeutic moiety used for ovarian cancer therapy. It is also developed by NeoPharm Labs using its proprietary NeoLipid^®^ technology. These liposomes are composed of a 90:5:5 molar ratio of DOPC, cholesterol and cardiolipin and a final total lipid to drug molar ratio of 33:1. This formulation achieved a maximum of 85% entrapment efficiency of PTX [130]. This technology eliminates the use of cremophor, which in turn circumvents the limitations and adverse effects associated with Taxol^®^ treatment. The clinical trial development of this formulation has been sponsored by Insys Therapeutics, Inc. and has an FDA-approved orphan drug designation for ovarian cancer therapy. Entrapment of PTX in liposomes demonstrated reduced toxicity with similar efficacy when compared with Taxol^®^. Pharmacokinetic data in patients are in correlation with preclinical data, which suggests comparable properties between LEP-ETU and Taxol^®^ [131,132]. The product is presently under phase II clinical trial investigation.

#### 4.2.8. Endotag-I

Endotag-I is another PTX liposomal formulation composed of cationic lipid dioleoyloxypropyltrimethylammonium (DOTAP) and neutral lipid (DOPC) [DOTAP:DOPC:PTX in 50:47:3]. This product has been developed by Medigene, which has an agreement with SynCore Biotechnology Co. for the complete technology transfer of EndoTAG^®^ [133,134]. These liposomal vesicles, being cationic in nature, interact with negatively charged endothelial cells required for tumour angiogenesis. PTX released from Endotag^®^-1 attacks the activated and dividing endothelial cells of a tumour, thus damaging the tumour blood supply without affecting healthy tissue. This mechanism of EndoTAG^®^ prevents angiogenesis in the tumour and thereby inhibits tumour growth. The anti-cancer efficacy of PTX, along with inhibition of tumour vasculature via vascular targeting of cationic liposomes, is the major mechanism behind the efficacy of EndoTAG^®^ in breast and pancreatic cancer therapy. The product is currently under phase II clinical trial investigation, where it showed prolonged survival rates when used along with gemcitabine in patients with pancreatic adenocarcinoma [135].

#### 4.2.9. Atragen^®^

Atragen^®^ is an all-trans retinoic acid encapsulated liposome composed of tretinoin, DMPC, and soybean oil with 2 mg of tretinoin/mL. All-trans retinoic acid is a protein synthesis inhibitor or retinoic acid receptor agonist that acts by affecting gene expression, leading to cell differentiation and a reduction in cell proliferation. Retinoic acid also inhibits telomerase, which leads to the shortening of telomeres that leads to apoptosis of tumour cells [136]. This product has been developed by Aronex Pharmaceuticals for acute promyelocytic leukaemia and other hematologic malignancies [137]. The encapsulation of retinoic acid into liposomes improves the half-life of intravenously administered tretinoin. The clinical development (Phase II) of Atragen^®^ has been designed to determine its potential towards management of hormone-resistant prostate cancer, renal cell carcinoma and acute myelogenous leukaemia [138].

### 4.3. Phase I

Apart from liposomal formulations listed under phase III and phase II trials, some liposomes like LEM-ETU, liposomal Grb-2, INX-0125, INX-0076 etc., are currently undergoing phase I clinical trial investigation. LEM-ETU is a liposomal formulation entrapped with Mitoxantrone, composed of a 90:5:5 molar ratio of DOPC, cholesterol and cardiolipin [139]. The product has been developed by NeoPharm’s NeoLipid liposome technology for the management of various cancers [140]. The co-lipid cardiolipin plays a key role in drug entrapment as this negatively charged diphosphatidyl glycerol lipid forms an electrostatic interaction with loaded moiety. This in turn leads to higher drug loading capability compared to other liposome formulations [141]. This product has been developed for the treatment of leukaemia, breast, stomach, liver and ovarian cancers. Liposomal Grb-2 is a liposomal formulation entrapped with antisense oligodeoxynucleotide growth factor receptor bound protein 2 (Grb-2). This protein possesses potent antineoplastic activity that acts by inhibition of cancer cell proliferation [142]. This has been developed by Bio-Path holdings for the treatment of breast cancer and various types of leukaemia. Overexpression of the Grb2 protein and amplification of the GRB2 gene have been reported in human cancer cell lines. Liposomal Grb-2, also known as BP-100-1.01, is a DOPC-incorporated antisense oligonucleotide formulation developed to inhibit the production of the growth factor receptor-bound protein-2 (Grb-2) [143]. The safety, maximum tolerated dose, optimal therapeutic dose and anticancer activity of liposomal Grb-2 have been investigated for the treatment of relapsed or refractory acute myeloid leukaemia. A dose range of 5 mg/m^2^ to 90 mg/m^2^ is well tolerated in these patients, with no product-related toxicities. Grb-2 target protein downregulation and promising anti-leukaemia activity were demonstrated in this study [144]. Liposomal formulations have been developed that incorporate sphingomyelin and saturated fatty acid chains in the cholesterol-rich liposomes [145].

INX-0125 (sphingosome-encapsulated vinorelbine tartrate) and INX-0076 (sphinogosome-encapsulated topotecan), which come under the category of sphingosomes, have been developed by Inex Pharmaceuticals and are undergoing phase I investigation. Both the liposome formulations are composed of cholesterol and SM in a 45:55 molar ratio and are indicated for advanced solid tumours [113]. INX-0125 has been developed for the treatment of Hodgkin’s disease and non-Hodgkin’s lymphoma. Sphingosomal encapsulation is a novel platform for targeted liposomal drug delivery, which is capable of increasing tumour targeting significantly and improves the duration of exposure for loaded anticancer agents. This also demonstrated an improved dose intensity without any increase in toxicity in preclinical trials [146]. Another product developed by the sphingosomal platform, INX-0076, encapsulates topotecan, which is a camptothecin analogue. This liposomal platform protects the drug from in vivo degradation, which has been demonstrated in preclinical trials and can specifically accumulate at target sites. This in turn increases the efficacy and reduces the dose compared to the free drug. Both the abovementioned sphingosomal formulations have been developed for advanced solid tumours [147].

Other products currently in phase I/II include PLK1 siRNA (TKM-080301) indicated for neuroendocrine tumours, PKN3 siRNA (Atu027) for pancreatic cancer and DOX (2B3-101) for solid tumours. TKM 080301 is a stable nucleic acid lipid particle (SNALP)-encapsulated siRNA targeting PLK1. This siRNA is targeted against polo-like kinase 1 (PLK1), a protein that plays a key role in tumour cell proliferation. Inhibition of PLK1 expression prevents tumour cell growth and thereby inhibits cancer cell proliferation. This product was evaluated in patients with gastrointestinal neuroendocrine tumours and adrenocortical carcinoma [148]. PKN3 siRNA is a lipoplex formulation containing siRNA directed against protein kinase N3 (PKN3). This is encapsulated in catiogenic and fusogenic lipids and demonstrated potential antineoplastic activity. Upon delivery, PKN3 siRNAs bind to PKN3 mRNAs and inhibit translation and expression of the PKN3 protein. Extensive preclinical studies demonstrated the anti-metastatic effect by targeting systemic vasculature in a prostate cancer model [149,150]. 2B3-101 is a PEGylated liposomal DOX formulation that targets patients with brain metastases [151]. Targeting brain tumours is challenging due to the limitation posed by the blood–brain barrier (BBB) as it restricts the entry of endogenous molecules as well as xenobiotics. The technology behind the development of 2B3-101 is termed G-technology, which is based on safe receptor biology in humans. It is the most flexible technology for the encapsulation of various molecules, i.e., high and low molecular weight drugs as well as hydrophilic and lipophilic compounds without any modifications of the pay load [152]. This product is developed based on glutathione PEGylation, where glutathione enhances the delivery of encapsulated moiety across the BBB [153].

Products including CEBPA siRNA (MTL-CEBPA) for liver cancer, Docetaxel (ATI-1123) for solid tumours, Vinorelbine (Alocrest) for breast and lung cancers, CPT (LiPlaCis) for advanced solid tumours, DOX (MCC-465) for metastatic stomach cancer, p53 gene (SGT-53) for various solid tumours etc., were also in phase I clinical trial investigation [154]. Other products currently undergoing phase I trials, their lipid compositions and indications are listed in Table 2.

## 5. Conclusions

Liposomes made their successful entry into the market in 1995 with the development of the PEGylated liposomal formulation Doxil^®^. Since its entry, there has been no looking back for these delivery systems, which have been explored for various diseases ranging from cancer treatment to pain management. The main advantages of liposomes include: control over pharmacokinetics’ and pharmacodynamics’ properties, improved bioavailability and limited toxicity. Together these confer on liposomes the ability to overcome the limitations of conventional therapy. Different types of liposomes, e.g., PEGylated liposomes (Lipodox), temperature sensitive liposomes (ThermoDox), cationic liposomes (EndoTAG-1) and liposomal vaccines (Epaxal and Inflexal V), demonstrate the intense research on liposomes. Several liposomes were successfully translated into the clinic and other liposomal formulations are in different phases of clinical investigation. Although many of these products have been proven to be beneficial in preclinical trials, only formulations that show efficacy in clinical trials will make their way into the clinic. In summary, the liposomes currently in clinical trials may provide benefits to the diversified patient population for various therapeutic applications.

## Figures and Tables

**Table 1:** Clinically used liposome-based products.

SN	Clinical Products (Approval Year)	Administration	Active Agent	Lipid/Lipid:Drug Molar Ratio	Indication	Company
1.	Doxil^®^ (1995)	i.v.	Doxorubicin	HSPC:Cholesterol:PEG 2000-DSPE (56:39:5 molar ratio)	Ovarian, breast cancer, Kaposi’s sarcoma	Sequus Pharmaceuticals
2.	DaunoXome^®^ (1996)	i.v.	Daunorubicin	DSPC and Cholesterol (2:1 molar ratio)	AIDS-related Kaposi’s sarcoma	NeXstar Pharmaceuticals
3.	Depocyt^®^ (1999)	Spinal	Cytarabine/Ara-C	DOPC, DPPG, Cholesterol and Triolein	Neoplastic meningitis	SkyPharma Inc.
4.	Myocet^®^ (2000)	i.v.	Doxorubicin	EPC:Cholesterol (55:45 molar ratio)	Combination therapy with cyclophosphamide in metastatic breast cancer	Elan Pharmaceuticals
5.	Mepact^®^ (2004)	i.v.	Mifamurtide	DOPS:POPC (3:7 molar ratio)	High-grade, resectable, non-metastatic osteosarcoma	Takeda Pharmaceutical Limited
6.	Marqibo^®^ (2012)	i.v.	Vincristine	SM:Cholesterol (60:40 molar ratio)	Acute lymphoblastic leukaemia	Talon Therapeutics, Inc.
7.	Onivyde™ (2015)	i.v.	Irinotecan	DSPC:MPEG-2000:DSPE (3:2:0.015 molar ratio)	Combination therapy with fluorouracil and leucovorin in metastatic adenocarcinoma of the pancreas	Merrimack Pharmaceuticals Inc.
8.	Abelcet^®^ (1995)	i.v.	Amphotericin B	DMPC:DMPG (7:3 molar ratio)	Invasive severe fungal infections	Sigma-Tau Pharmaceuticals
9.	Ambisome^®^ (1997)	i.v.	Amphotericin B	HSPC:DSPG:Cholesterol:Amphotericin B (2:0.8:1:0.4 molar ratio)	Presumed fungal infections	Astellas Pharma
10.	Amphotec^®^ (1996)	i.v.	Amphotericin B	Cholesteryl sulphate:Amphotericin B (1:1 molar ratio)	Severe fungal infections	Ben Venue Laboratories Inc.
11.	Visudyne^®^ (2000)	i.v.	Verteporphin	Verteporphin:DMPC and EPG (1:8 molar ratio)	Choroidal neovascularisation	Novartis
12.	DepoDur™ (2004)	Epidural	Morphine sulfate	DOPC, DPPG, Cholesterol and Triolein	Pain management	SkyPharma Inc.
13.	Exparel^®^ (2011)	i.v.	Bupivacaine	DEPC, DPPG, Cholesterol and Tricaprylin	Pain management	Pacira Pharmaceuticals, Inc.
14.	Epaxal^®^ (1993)	i.m.	Inactivated hepatitis A virus (strain RGSB)	DOPC:DOPE (75:25 molar ratio)	Hepatitis A	Crucell, Berna Biotech
15.	Inflexal^®^ V (1997)	i.m.	Inactivated hemaglutinine of Influenza virus strains A and B	DOPC:DOPE (75:25 molar ratio)	Influenza	Crucell, Berna Biotech

i.v. (intravenous); i.m. (intramuscular); HSPC (hydrogenated soy phosphatidylcholine); PEG (polyethylene glycol); DSPE (distearoyl-sn-glycero-phosphoethanolamine); DSPC (distearoylphosphatidylcholine); DOPC (dioleoylphosphatidylcholine); DPPG (dipalmitoylphosphatidylglycerol); EPC (egg phosphatidylcholine); DOPS (dioleoylphosphatidylserine); POPC (palmitoyloleoylphosphatidylcholine); SM (sphingomyelin); MPEG (methoxy polyethylene glycol); DMPC (dimyristoyl phosphatidylcholine); DMPG (dimyristoyl phosphatidylglycerol); DSPG (distearoylphosphatidylglycerol); DEPC (dierucoylphosphatidylcholine); DOPE (dioleoly-sn-glycero-phophoethanolamine).

**Table 2:** Liposomal formulations present in clinical trials.

SN	Products	Administration	Active Agent	Lipid Composition	Indication	Company
Phase III						
1.	Arikace	Aerosol delivery	Amikacin	DPPC and cholesterol	Lung infections	Transave Inc.
2.	Stimuvax	s.c.	Tecemotide	Cholesterol, DMPG, DPPC	Non-small cell lung cancer	Oncothyreon Inc.
3.	T4N5 liposomal lotion	Topical	T4 endonuclease V	Egg lecithin	Xeroderma pigmentosum	AGI Dermatics Inc.
4.	Liprostin	i.v.	Prostaglandin E-1 (PGE-1)	Unknown	Restenosis after angioplasty	Endovasc Inc.
5.	ThermoDox	i.v.	Doxorubicin	DPPC, Myristoyl stearyl phosphatidylcholine and DSPE-*N*-[amino(polyethylene glycol)-2000]	Hepatocellular carcinoma and also recurring chest wall breast cancer	Celsion
6.	Lipoplatin	i.v.	Cisplatin	DPPG, soy phosphatidyl choline, mPEG-distearoyl phosphatidylethanolamine lipid conjugate and cholesterol	Non-small cell lung cancer	Regulon Inc.
Phase II						
7.	Aroplatin	i.v.	Platinum analogue cis-(trans- R,R-1,2-diaminocyclohexane) bis (neodecanoato) platinum (II)	DMPC and DMPG	Metastatic colorectal cancer	Agenus Inc.
8.	Liposomal annamycin	i.v.	Semi-synthetic doxorubicin analogue annamycin	DMPC and DMPG	Relapsed or refractory acute myeloid leukaemia	Aronex Pharmaceuticals
9.	SPI-077	i.v.	Cisplatin	Soybean phosphatidylcholine, cholesterol	Lung, head and neck cancer	Alza Corporation
10.	OSI-211	i.v.	Lurtotecan	HSPC and cholesterol	Ovarian, head and neck cancer	OSI Pharmaceuticals
11.	S-CKD602	i.v.	Potent topoisomerase I inhibitor	Phospholipids covalently bound to mPEG	Cancer	Alza Corporation
12.	LE-SN38	i.v.	Irinotecan’s active metabolite	DOPC, cholesterol and cardiolipin	Advanced colorectal cancer	NeoPharm Labs Ltd.
13.	LEP-ETU	i.v.	Paclitaxel	DOPC, cholesterol and cardiolipin	Cancer	NeoPharm Labs Ltd.
14.	Endotag-I	i.v.	Paclitaxel	DOTAP: DOPC: Paclitaxel	Breast and pancreatic cancers	Medigene
15.	Atragen	i.v.	All-trans retinoic acid	DMPC and soybean oil	Hormone-resistant prostate cancer, renal cell carcinoma and acute myelogenous leukaemia	Aronex Pharmaceuticals
Phase I						
16.	LEM-ETU	i.v.	Mitoxantrone	DOPC, cholesterol and cardiolipin	Various cancers	NeoPharm Labs Ltd.
17.	Liposomal Grb-2	i.v.	Antisense oligodeoxynucleotide growth factor receptor bound protein 2 (Grb-2)	Unknown	Hematologic malignancies	Bio-Path holdings
18.	INX-0125	i.v.	Vinorelbine tartrate	Cholesterol and sphingomyelin	Advanced solid tumours	Inex Pharmaceuticals
19.	INX-0076	i.v.	Topotecan	Cholesterol and sphingomyelin	Advanced solid tumours	Inex Pharmaceuticals
20.	TKM-080301	Hepatic intra-arterial administration	PLK1 siRNA	Unique LNP technology (formerly referred to as stable nucleic acid-lipid particles or SNALP)	Neuroendocrine tumours	Tekmira Pharmaceuticals
21.	Atu027	i.v.	PKN3 siRNA	AtuFECT01	Pancreatic cancer	Silence Therapeutics
22.	2B3-101	i.v.	Doxorubicin	Glutathione PEGylated liposomes	Solid tumours	2-BBB therapeutic
23.	MTL-CEBPA	i.v.	CEBPA siRNA	SMARTICLES^®^ liposomal nanoparticles	Liver cancer	MiNA Therapeutics
24.	ATI-1123	i.v.	Docetaxel	Protein stabilizing liposomes (PSL™)	Solid tumours	Azaya therapeutic
25.	LiPlaCis	i.v.	Cisplatin	The lipid composition of the LiPlasomes is tailored to be specifically sensitive to degradation by the sPLA2 enzyme	Advanced solid tumours	Oncology Venture
26.	MCC-465	i.v.	Doxorubicin	DPPC, cholesterol and maleimidated palmitoyl phosphatidyl ethanolamine; immunoliposomes tagged with PEG and the F(ab′)2 fragment of human monoclonal antibody GAH	Metastatic stomach cancer	Mitsubishi Tanabe Pharma Corporation
27.	SGT-53	i.v.	p53 gene	Cationic lipids complexed with plasmid DNA encoding wild-type p53 tumour suppressor protein	Various solid tumours	SynerGene Therapeutics
28.	Alocrest	i.v.	Vinorelbine	Sphingomyelin/cholesterol (OPTISOME™)	Breast and lung cancers	Spectrum Pharmaceuticals

DMPG (Dimyristoyl phosphatidylglycerol); DPPC (Dipalmitoyl phosphatidylcholine); DPPG (Dipalmitoyl phosphatidylglycerol); DMPC (dimyristoyl phosphatidylcholine); HSPC (hydrogenated soy phosphatidylcholine); PEG (polyethylene glycol); mPEG (methoxy polyethylene glycol); DOPC (dioleoylphosphatidylcholine); DSPE (distearoyl-sn-glycero-phosphoethanolamine); i.v. intravenous.
